# Cutaneous Ulcers in an Untreated HIV Patient

**DOI:** 10.5826/dpc.1102a07

**Published:** 2021-03-08

**Authors:** Xavier Bosch-Amate, Xavier Fustà-Novell, Daniel Morgado-Carrasco

**Affiliations:** 1 Department of Dermatology, Hospital Clínic de Barcelona, Universitat de Barcelona, Spain

**Keywords:** cutaneous ulcers, syphilis, malignant syphilis, men who have sex with men

## Case Presentation

**Figure 1:**
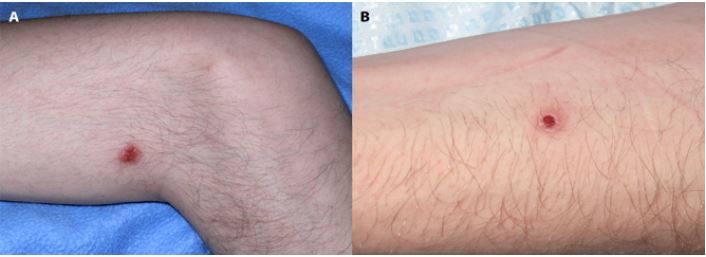
(A, B) Malignant syphilis. Erythematous and ulcerated plaques with well-demarcated borders on the left arm.

A 22-year-old man presented with a 6-month history of asthenia, anorexia, severe weight loss of 20 kg, low-grade fever, and cutaneous lesions that had occurred within 1 month. More importantly, this patient presented with untreated HIV infection. Physical examination revealed a few erythematous and ulcerated plaques (some with necrotic crusts) on his trunk and extremities (Figure 1) and absence of lesions on mucous membranes, palms and soles. Blood tests showed 284 CD4/μL and HIV viral load 243,000 copies/mL. Nontreponemal tests showed elevated VDRL titers (1/32). Lumbar puncture ruled out neurosyphilis. With a diagnosis of malignant syphilis, benzathine penicillin 2.4 MU was administered intramuscularly with complete resolution of symptoms.

## Teaching Point

Syphilis has a broad spectrum of mucocutaneous manifestations. Malignant syphilis is a rare form of secondary syphilis, associated in most cases with HIV infection, that could present with a few cutaneous lesions. In our case histopathology was not done; however, it usually shows the presence of a dense inflammatory infiltrate, sometimes with a lichenoid pattern, with lymphocytes, plasma cells, and occasional presence of granulomas. Early detection of the coexistence of secondary syphilis and HIV infection is essential for correct management and the avoidance of serious complications [1,2].
